# A Study of the Factors Impeding Proper Dietary Habits: An Investigation Using the Japanese Comprehensive Survey of Living Conditions

**DOI:** 10.3390/clinpract14060176

**Published:** 2024-10-23

**Authors:** Akira Komatsuzaki, Sachie Ono, Kanako Mitomi, Kiyoka Arashi, Yukika Enoki, Kanako Seino, Naru Komatsuzaki, Yuuko Ikeda

**Affiliations:** 1 Department of Dental Hygiene, The Nippon Dental University, College at Niigata, 1-8 Hamaura cho, Chuo-ku, Niigata 951-8580, Japan; kiyoka@ngt.ndu.ac.jp (K.A.); enoki11@ngt.ndu.ac.jp (Y.E.); springsea@ngt.ndu.ac.jp (K.S.); ikeday@mx.ngt.ndu.ac.jp (Y.I.); 2 Department of Preventive and Community Dentistry, School of Life Dentistry at Niigata, The Nippon Dental University, 1-8 Hamaura-cho, Chuo-ku, Niigata 951-8580, Japan; sachie@ngt.ndu.ac.jp; 3 Laboratory of Dental Technology, The Nippon Dental University, Niigata Hospital, 1-8 Hamaura cho, Chuo-ku, Niigata 951-8580, Japan; kmitomi@ngt.ndu.ac.jp

**Keywords:** diet, dietary management skill, lifestyle-related diseases, lifestyle habits, symptom

## Abstract

(1) Background: Diet is significant for nutritional intake and serves as an essential element for improving quality of life (QOL). Poor dietary management skills increase the risk of onset or progression of lifestyle-related diseases, and, in particular, are a factor in reduced QOL during old age. This study aimed to clarify the physical and social background factors impeding dietary self-management. (2) Methods: The study participants were 3814 men (age range, 30–69 years) extracted from anonymous data comprising 15,294 persons provided from the Japanese national statistics database. The participants were classified into two groups (Concerned vs. Unconcerned) according to whether they were concerned about their diet. Adjusted odds ratios (ORs) for diet-conscious behaviors were then obtained by means of binomial logistic regression analysis performed following univariate analysis. (3) Results: The Concerned and Unconcerned groups comprised 2548 (66.8%) and 1266 subjects (33.2%), respectively. The diet-conscious behavior with the highest response rate was eating regularly (46.7%). The most frequent items in the Unconcerned group were the subjective symptom “irritable” (48.9%), high stress (46.3%), working more than 56 h/week (43.8%), and smoking (41.9%). The only item with a large significant OR in the binomial logistic regression analysis was smoking (OR: 2.2). (4) Conclusions: These results suggest that a smoking habit and stress are factors that impede diet management behaviors.

## 1. Introduction

Eating is vital in the sense that it provides not only the nutrients needed to sustain life, but also an everyday pleasure that is a fundamental factor in improving the quality of life (QOL) [1]. In its Global Strategy on Diet, Physical Activity and Health (DPAS) in 2004 [2], the World Health Organization (WHO) recommended that the risk factors for lifestyle diseases caused by an unhealthy diet be reduced through health projects [3]. In addition, as a specific activity, the WHO member states have aimed for a 30% reduction in salt intake [4], and in 2023, the WHO published new guidelines for reviewing unhealthy diets with the aim of reducing the number of patients with high blood pressure [5].

In Japan, it has been reported that a deterioration in the ability to manage one’s own diet is a risk factor for the onset and progression of lifestyle-related diseases and a range of health disorders [6]. The National Health and Nutrition Survey points to skipping breakfast, insufficient fruit and vegetable intake, and excessive salt intake as specific problems in the diet of the Japanese people [7]. The number of cases of obesity due to overeating has increased, and the government has been implementing specific health checkups and health guidance nationwide since 2008 as measures to combat metabolic syndrome [8].

At the same time, excessive dieting among young women [9] and undernourishment among older adults [10] are also problems, and the importance of nutritional and dietary guidance that suits the health status of the individual is regarded as a top priority in health education. The Basic Act on “*Shokuiku*” (Food and Nutrition Education) has been established, emphasizing the importance of guidance from early childhood while also bolstering diet and nutrition guidance based on the home and community for adults from the perspective of preventing the need for care in later life [11].

In the field of dentistry in particular, guidance relating to the formation of proper dietary habits is being given with the aim of preventing malnutrition during old age, when there is increased tooth loss [12]. This dietary guidance takes a range of approaches, including improved denture function [13], the promotion of home dental care [14], training in ingestion and swallowing [15], and the prevention of oral dryness [16]. In the medical field, dietary therapy is regarded as a part of whole-body medical management in the treatment of diseases such as diabetes [17], and it is possible to aim for disease improvement outside of hospitalization through appropriate diet self-management and the formation of proper dietary habits.

However, appetite control is an extremely complex physiological mechanism [18], and an individual’s social environment can impede the formation of proper dietary habits. It may therefore be surmised that clarifying the background factors in the formation of proper dietary habits will not be straightforward.

Here, we focused on the Comprehensive Survey of Living Conditions, which can be used as a largescale database, to obtain basic data on dietary management from anonymized data analysis. The most recent Comprehensive Survey of Living Conditions looked at the importance of dietary management behavior, with the inclusion of four specific items: eating regularly, nutritional balance, salt intake, and concern for overeating [19]. A detailed health form is also included every 3 years when the largescale survey is conducted. We considered that it would be possible to clarify the factors that impede dietary management behaviors from the data on physical and social factors obtained from the Comprehensive Survey of Living Conditions.

To date, we have reported the results of numerous analyses of the Comprehensive Survey of Living Conditions, which include a wide range of data on subjective symptoms, hospital visits, and lifestyle habits [20,21,22]. In the present study, we made use of the largescale database to analyze the causal structure of the background factors for dietary management behavior.

## 2. Materials and Methods

### 2.1. Study Design and Subject Data

**Figure 1:**
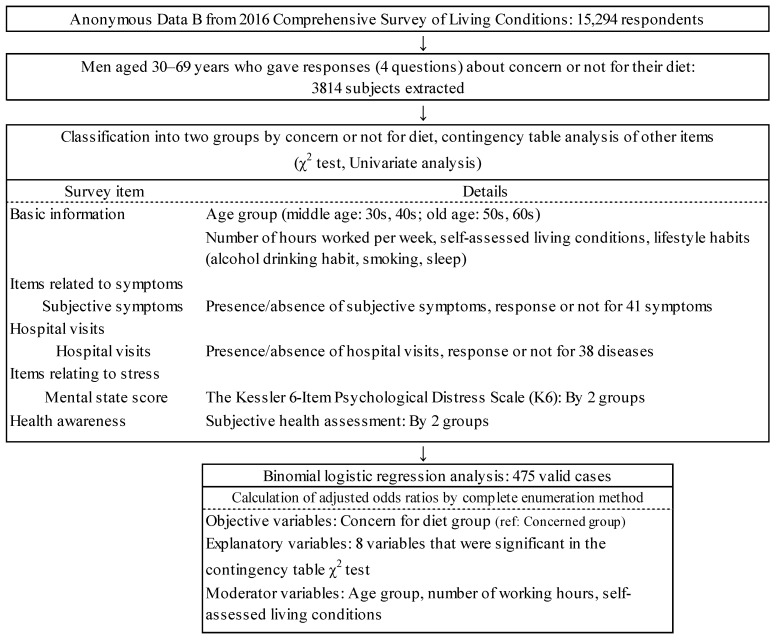
Data flow for the analysis of anonymous data.

This study was conducted using a cross-sectional design. For the subjects (anonymous individual data), anonymous data files for 15,294 people were obtained from the Comprehensive Survey of Living Conditions with the permission of the Ministry of Health, Labour and Welfare (MHLW), and stepwise sampling was performed as shown in Figure 1. The subjects extracted for the univariate analysis (contingency table analysis) were 3814 men (age range, 30–69 years) who responded to the four items relating to whether they were or were not concerned about their diet. The subject data were analyses from the household survey (survey of sex, age, household economic consciousness, etc.) and the health survey (survey of symptoms, hospital visits, diseases, lifestyle habits, health awareness, etc.) in accordance with the stepwise procedure shown in Figure 1.

### 2.2. Classification into Groups Concerned or Not About Diet and Contingency Table Analysis (Univariate Analysis) of Survey Items

The subjects were classified into two groups according to their responses to the following four items relating to their concern for their diet: eat regularly, eat a proper nutritional balance, eat lightly seasoned foods, and avoid overeating. Those who responded “Yes” to any of the four items were classified into the Concerned group (2548 subjects, 66.8%), and those who responded “No” to all four items were classified into the Unconcerned group (1266 subjects, 33.2%) (Table 1). The responses to the other items in the survey were then compared between the two diet concern groups.

In a univariate analysis, the proportion of each group responding to the survey items was compared by means of a contingency table (χ^2^ test, univariate odds ratio [OR] and 95% confidence interval) to check for associations.

### 2.3. Comparison of Subjective Symptom and Disease Response Rate Rankings by Concern for Diet

The rankings of the response rates for symptoms and diseases were compared between the Concerned and Unconcerned groups by means of the Wilcoxon signed-rank test (symptoms or diseases with a response rate of ≥5% were tested).

### 2.4. Analysis of the Degree of Influence on the Concern for Diet by Multivariate Analysis (Binomial Logistic Regression Analysis)

A binomial logistic regression analysis (complete enumeration method) was carried out with symptoms or diseases shown by the contingency table analysis to have an association with concern for diet, together with moderator variables such as gender and working hours as explanatory variables and concern for diet (reference: Concerned group) as objective variables, and adjusted ORs were calculated.

### 2.5. Statistical Analysis

Basic data aggregation was carried out using Excel (Microsoft Japan, Tokyo, Japan). The χ^2^ test, calculation of univariate ORs, Wilcoxon signed-rank test, and binomial logistic regression analysis were carried out using BellCurve for Excel 2019MSO (BellCurve, Tokyo, Japan). The level of significance was set at *p* < 0.05 for all statistical tests.

### 2.6. Ethical Considerations

The present study was approved by the Ethical Review Board of the Nippon Dental University College at Niigata (No. NDUC-106) and the MHLW (Government Statistics 0805 No. 3) on the basis of Article 36 of the Statistics Act. This study was conducted in accordance with the Declaration of Helsinki, with measures for the protection of personal information in line with the ethical principles for epidemiological studies set out by the Ministry of Education, Culture, Sports, Science and Technology and the MHLW of Japan. The authors used data files in tabular form that were provided by the MHLW following anonymization, and all data comprised only responses from survey participants who had given their informed consent.

## 3. Results

### 3.1. Classification into Groups by Concern for Diet

Of the subjects, 2548 (66.8%) responded to at least one of the four items regarding concern for diet and were classified into the Concerned group, accounting for two-thirds of the total. Of the four items, the highest response rate was for “eat regularly” (1781, 46.7%), and the lowest response rate was for “eat lightly seasoned foods” (780, 20.5%) (Table 1).

Subjects who did not respond to any of the items were classified into the Unconcerned group (1266, 33.2%). The following univariate and multivariate analyses were conducted according to this grouping.

### 3.2. Results of Contingency Table Analysis of Items Related to Lifestyle, Health, and Lifestyle Habits by Diet Concern Group

The results of the univariate analysis of age, number of hours worked, self-assessed living conditions (economic environment), and lifestyle habits are shown in Table 2. As an overall trend, there was a tendency toward a greater number of favorable responses in the Concerned group, and for all items except drinking alcohol, there was a significant difference (*p* < 0.01) between the two diet concern groups in the proportion of respondents (Table 2).

For variables other than age, the univariate ORs in order of size were smoking habit (1.87), mental state score (1.74), and sleeping time (1.66), and all ORs other than alcohol consumption were significant.

### 3.3. Results of Contingency Table Analysis of Subjective Symptoms and Items Related to Hospital Visits by Diet Concern Group

Table 3 shows the results for the presence of subjective symptoms and the two symptoms (irritable, headache) found to have significant differences in response rates in the contingency table analysis of different symptoms.

There was a tendency for approximately 3% more subjects in the Concerned group to respond that they had subjective symptoms compared with the Unconcerned group, while a significantly higher (*p* < 0.01) proportion of subjects (approximately 50%) responded to “irritable” and “headache” in the Unconcerned group.

Table 3 shows the results for the presence of hospital visits and one disease found to have significant differences in response rates in the contingency table analysis of diseases responsible for hospital visits (presence of hospital visits: *p* < 0.01, high blood pressure: *p* < 0.05). The proportion of subjects with hospital visits was higher in the Concerned group, and the OR was significant, with a value < 1, which is the opposite of the results for subjective symptoms. The proportion of subjects responding to high blood pressure was more than 5% higher than those not responding in the Concerned group. Among the items relating to concern for diet, eating lightly seasoned foods accounted for a high proportion (38.2%) of subjects with high blood pressure, and this appears to be a factor for the high proportion of subjects with high blood pressure in the Concerned group.

### 3.4. Comparison of Subjective Symptom and Disease Response Rate Rankings by Diet Concern Group

Table 4 shows the top five symptoms ranked for response rate by diet concern group. Both groups showed the same symptoms in first and second places, but a difference was found from third place onward, with dental symptoms appearing in the Concerned group. The Wilcoxon signed-rank test was carried out on the order of the 29 symptoms with a response rate of ≥1%, and a significant difference (*p* < 0.01) was found between the two groups.

Table 4 shows the top five diseases ranked for response rate by diet concern group. The top five diseases are the same in both groups, and in the same order, and both groups have dental disease in fourth place. A tendency toward differences in the ranking was observed from fifth place onward, with the Unconcerned group including items such as allergic rhinitis and depression or other diseases in the top 10. The Wilcoxon signed-rank test was carried out on the order of the 26 diseases with a response rate of ≥0.5%, and a significant difference (*p* < 0.01) was found between the two groups.

### 3.5. Results of Multivariate Analysis with Diet Concern Group as the Objective Variable

Table 5 shows the results of multivariate analysis. A multivariate analysis (binomial logistic regression analysis) was conducted by constructing an analytic model with the factors assumed to affect the consideration for diet groups based on the results of the univariate analysis by contingency table (Table 2 and Table 3). The objective variable was set at 1 for the Unconcerned group, with the Concerned group as the control (0). Age and number of hours worked were inserted as moderator variables, and the adjusted ORs thus obtained were compared.

Because the purpose of this analytical model was to examine the effect of factors that impede diet-conscious behaviors, a higher OR indicated a greater effect as an impeding factor. The adjusted OR was significant only for smoking (OR: 2.2). Symptoms for which the OR was not significant but >1 included headache, and the OR for headache in the univariate analysis was significant, suggesting that this may be a factor impeding diet-conscious behaviors.

Conversely, the disease high blood pressure was included in the explanatory variables with an OR < 1, and its OR was also <1 in the univariate analysis, suggesting that it may be a factor motivating diet-conscious behaviors.

## 4. Discussion

According to the results of this study, the items with a high proportion of responses in the Unconcerned group included “irritable” in the subjective symptoms (48.9%), high stress on the K6 (46.3%), and working > 56 h per week (43.8%).

There have been numerous reports that stress and the working environment affect diet and nutritional intake [23,24,25], and it has also been reported that a high stress load is a risk factor for gastrointestinal disease [26]. With regard to concern for diet, which is the focus of the present study, Sogari G. et al. [27] reported an association between regular dietary intake and stress. Other than this, however, there are only scattered reports, such as one by Zahedi et al. [28] analyzing the relationship between skipping breakfast and mental state, and, with the exception of the regulation of salt intake as medical management for patients with high blood pressure [29], concern for diet is not a research topic that has received much attention. In a prior study, we analyzed the association of stress with subjective symptoms and diseases [20], but we did not analyze considerations of dietary intake. Stress is a background factor for lifestyle-related diseases, and analyses of the effects of stress are important for countries carrying out health promotion activities that emphasize measures for lifestyle-related diseases.

Similarly, the results of this study did not reveal an association between drinking or smoking and diet concern group. It is possible that the answers to these negative habit questions are inaccurate, and further investigation is required [30].

In the present study, >60% of the subjects were concerned about their eating behavior, suggesting that this is an important research topic from the perspectives of nutritional guidance and QOL. An association with concern for diet was found with some subjective symptoms and diseases, indicating that guidance that aims for the formation of healthy eating habits is also important.

This point of view is not intended to criticize the trend in recent years for dietary guidance to be oriented toward metabolic syndrome measures [8]; rather, the results of the present study lend support to the idea of concern for diet being reflected in metabolic syndrome measures.

At the same time, the results of the multivariate analysis in the present study suggest that improved management skills with regard to diet may also be expected to have effects on the control of negative health habits such as smoking. A possible reason for this is that placing emphasis on the timing and content of meals may lead to a comparative reduction in the time and value of smoking and its psychological dependence. It has been reported that with comprehensive guidance for quitting smoking in a closed environment, greater importance came to be placed on the formation of other health habits, including diet [31].

In addition, it should be noted that dental symptoms and dental disease were among the highest-ranked symptoms and diseases in the comparison of response rankings between the two diet concern groups, and in particular, dental symptoms ranked high in the Unconcerned group. This suggests the possibility that the tendency to neglect untreated teeth may coincide with the tendency to disregard diet, which is consistent with the trend found among young people by Shizuma et al. [32]. Further scrutiny from the point of view of dentistry is needed in the future.

The increase in eating out and the Westernization of the Japanese diet in recent years are often viewed as problematic trends in Japanese dietary intake. It has been pointed out that it can be difficult to curb eating out, which often involves excessive calorie intake [33]. It is perhaps important to provide guidance in concern for diet covering the right way to eat to people who eat out frequently. The merit of this paper is primarily that it is focused on the existence of factors that can impede diet-conscious behaviors. Changes in dietary habits can affect health status, and eating habit guidance can also regulate the development of metabolic diseases [34]. The most important message seems to be that data from research on this field in Japan have not been sufficient in either quantity or quality for realizing a healthy society.

Raine et al. [35] analyzed background factors for a healthy diet in Canada and found that groups such as family and friends have a large influence; they also noted social and cultural influences. In Japan, it is common for families to teach their children table manners based on Japanese-style food, and it may be surmised that an individual’s family will have a large effect on their eating behavior. In addition, shokuiku (food and nutrition education) school-based programs that teach diet and nutrition have become widespread in recent years in Japan [36,37], and a study of university students reported that the group that lacked understanding of shokuiku had a higher risk of untreated caries than the group that knew about it [38]. This suggests that consideration of diet can affect dental and other types of health.

The comparisons of disease and symptom rankings in the present study showed that hospital visits for dental symptoms and diseases ranked highly, which is likely due to the effects of eating snacks and caries onset as a result of sustained poor oral hygiene. Additionally, the fact that fatigue-related symptoms and diseases were high on the list also suggests that they may be risk factors for dietary disorders and malnutrition.

However, the Comprehensive Survey of Living Conditions does not carry out a full-body examination that includes the mouth, so there are limits to the inferences that can be drawn based on this survey alone. We anticipate that our hypothesis will be supported by the publication of reports on the outcomes of local shokuiku activities in the future.

It is becoming increasingly difficult for families to eat together in present-day Japan because of changes in family lifestyles, such as the increases in dual-income households [39], children attending cram schools [40], and poverty [41]. Because it may be conjectured that dietary habits cannot easily be improved after adulthood, it is important to continue dietary guidance during adolescence through extensive public education on the importance of the effects of proper eating at opportune times, such as before job-hunting.

This study has some limitations. First, concern for diet was surveyed by means of a questionnaire that relied on subjective assessment, and thus, there are reservations about the effects of bias. Second, this study was an analysis of the results of a cross-sectional survey, and because it is difficult to verify causal relationships, the results may have been influenced by unknown confounding factors that were not foreseen. As anonymous data from a national statistics survey were used, the questions are limited, and it is, therefore, difficult to optimize the content and structure of the questions.

In particular, in this cross-sectional study, symptoms and disease were simultaneously analyzed as independent variables, which can be either cause or effect in terms of time, so caution must be taken in interpreting effects.

Furthermore, the adjustment variables adopted in the analytical model to control confounding factors were entered at the discretion of the authors, and the judgment may differ depending on the analyst. There are many factors that are reflected in dietary habits, and it is assumed that in addition to demographic variables, it is also necessary to consider the dietary habits of the family.

Moreover, although this study only targeted men, in Japan, where the working environment for men and women is different [42], it is necessary to focus on the influence of gender, and we would like to clarify the influence of gender differences in the next study. We hope that we can overcome these difficulties by conducting repeated, multifaceted analyses to clarify the factors involved in diet from a comprehensive viewpoint that includes dentistry, gender, and health habits in the future.

In addition, this study did not include any investigations from the point of view of nutrition. There is a need to carry out an investigation of factors associated with dietary habits using other national statistics data on nutrition.

## 5. Conclusions

In this study, we examined the association between subjective symptoms and diseases and the presence or otherwise of concern for diet based on the results of the Comprehensive Survey of Living Conditions.

We were able to confirm symptoms and diseases that are inferred to have an association with concern for diet management behaviors, as well as the association between working time, smoking, and stress. That fatigue-related symptoms and diseases were high on the list also suggests that they may be risk factors for dietary disorders and malnutrition.

These results suggest the existence of factors that can impede diet-conscious behaviors. Therefore, it is important to evaluate concern for dietary intake in future measures against lifestyle-related diseases during adulthood.

## Figures and Tables

**Table 1:** Group classification by response to 4 items relating to concern for diet.

Response Item	Concern for Diet (4 Items)				Classification into 2 Groups by Concern for Diet
	Eat Regularly	Eat Proper Nutritional Balance	Eat Lightly Seasoned Foods	Avoid Overeating	
					Performed any of the 4 items: Concerned group
Yes	1781 (46.7)	1257 (33.0)	780 (20.5)	1297 (34.0)	2548 (66.8)
					Indifferent to any of the items: Unconcerned group
No	2033 (53.3)	2557 (67.0)	3034 (79.5)	2517 (66.0)	1266 (33.2)
Total	3814 (100.0)	3814 (100.0)	3814 (100.0)	3814 (100.0)	3814 (100.0)
					No. of persons (%)

**Table 2:** Responses relating to lifestyle, health, and lifestyle habits by diet concern group.

Item	Response Category (*)	Unconcerned Group (1)		Concerned Group (0)		Total		*p*-Value (χ^2^ Test)	Unadjusted Odds (95% CI Range)
Age group	30–49 years (1)	742	(43.3)	971	(56.7)	1713	(100.0)	0.001 **	2.30
	50–69 years (0)	524	(24.9)	1577	(75.1)	2101	(100.0)		(2.00–2.64)
Working time	≥56 h (1)	246	(43.8)	316	(56.2)	562	(100.0)	0.001 **	1.62
	<56 h (0)	823	(32.5)	1713	(67.5)	2536	(100.0)		(1.35–1.95)
Self-assessed living conditions	Difficult (1)	805	(36.8)	1382	(63.2)	2187	(100.0)	0.001 **	1.47
	Normal/comfortable (0)	461	(28.3)	1166	(71.7)	1627	(100.0)		(1.28–1.69)
Subjective health assessments	Not good, not very good (1)	162	(38.8)	255	(61.2)	417	(100.0)	0.009 **	1.32
	Normal/good (0)	1098	(32.5)	2282	(67.5)	3380	(100.0)		(1.07–1.63)
Mental health score (K6)	≥10 (1)	149	(46.3)	173	(53.7)	322	(100.0)	0.001 **	1.74
	≤9 (0)	1175	(33.1)	2375	(66.9)	3550	(100.0)		(1.38–2.19)
Sleeping time	<6 h (1)	586	(40.2)	870	(59.8)	1456	(100.0)	0.001 **	1.66
	≥6 h (0)	678	(28.8)	1675	(71.2)	2353	(100.0)		(1.45–1.91)
Alcohol drinking habit	Yes	474	(31.9)	1012	(68.1)	1486	(100.0)	0.266	0.92
	No	774	(33.6)	1527	(66.4)	2301	(100.0)		(0.80–1.06)
Smoking habit	Yes (1)	581	(41.9)	806	(58.1)	1387	(100.0)	0.001 **	1.87
	No (0)	668	(27.9)	1730	(72.1)	2398	(100.0)		(1.62–2.15)

* Variables for the binomial logistic regression analysis. Number of persons (%), **: *p* < 0.01.

**Table 3:** Responses to subjective symptoms and disease by diet concern group.

Item	Response Category (*)	Unconcerned Group (1)		Concerned Group (0)		Total		*p*-Value (χ^2^ Test)	Unadjusted Odds (95% CI Range)
Subjective symptoms	Yes	377	(35.5)	685	(64.5)	1062	(100.0)	0.067	1.15
	No	884	(32.4)	1846	(67.6)	2730	(100.0)		(0.99–1.33)
Irritable	Yes (1)	46	(48.9)	48	(51.1)	94	(100.0)	0.001 **	1.84
	No (0)	331	(34.2)	637	(65.8)	968	(100.0)		(1.20–2.82)
Headache	Yes (1)	47	(50.5)	46	(49.5)	93	(100.0)	0.002 **	1.98
	No (0)	330	(34.1)	639	(65.9)	969	(100.0)		(1.29–3.03)
Hospital visits	Yes	396	(25.9)	1131	(74.1)	1527	(100.0)	0.001 **	0.57
	No	862	(38.0)	1409	(62.0)	2271	(100.0)		(0.50–0.66)
High blood pressure	Yes (1)	110	(22.2)	385	(77.8)	495	(100.0)	0.022 *	0.75
	No (0)	286	(27.7)	746	(72.3)	1032	(100.0)		(0.58–0.96)

* Variables for the binomial logistic regression analysis. Number of persons (%) **: *p* < 0.01, *: *p* < 0.05.

**Table 4:** Response rate ranking for symptoms and diseases (up to 5th place) by diet concern group.

Order	Symptoms				Diseases			
	Unconcerned Group	%	Concerned Group	%	Unconcerned Group	%	Concerned Group	%
1st place	Lower back pain	36.3	Lower back pain	40.1	High blood pressure	27.8	High blood pressure	34
2nd place	Stiff shoulders	31.3	Stiff shoulders	28.3	Diabetes	14.4	Diabetes	17.2
3rd place	Lethargic	18.6	Cough, phlegmatic	15.3	Hyperlipidemia	13.9	Hyperlipidemia	13.9
4th place	Cough, phlegmatic/joint pain in hands/feet	15.1	Dental symptoms	14.3	Dental disease	12.4	Dental disease	12.9
5th place	Numb limbs	14.9	Lethargic	14.2	Lower back pain	12.1	Lower back pain	10.5
Wilcoxon signed-rank test *	*p* < 0.01				*p* < 0.01			

(* NB: 29 symptoms, 26 diseases were tested).

**Table 5:** Results of binomial logistic regression analysis.

Objective Variable	Explanatory Variable	Partial Regression Coefficient	Odds Ratio	95% CI Lower Value	95% CI Upper Value	Wald	*p*-Value
Concern for diet group	Smoking	0.79	2.20	1.43	3.40	12.81	<0.001 **
	Working time	0.49	1.63	0.97	2.75	3.35	0.066
	Headache	0.36	1.43	0.69	2.97	0.94	0.331
	Sleeping time	0.31	1.36	0.89	2.07	2.09	0.147
	Mental health score (K6)	0.23	1.26	0.68	2.33	0.55	0.458
	Self-assessed living conditions	0.02	1.02	0.66	1.58	0.01	0.904
	High blood pressure	−0.21	0.80	0.47	1.35	0.66	0.414
	Irritable	−0.23	0.78	0.34	1.80	0.31	0.573
	Age	−0.63	0.52	0.33	0.82	7.73	0.005 **

Coefficient of determination (Cox–Snell) 0.09, likelihood ratio test: *p* < 0.001. Subjective health assessments and hospital visits were excluded from the analysis: linear combination. **: *p* < 0.01.

## Data Availability

The original contributions presented in the study are included in the article, further inquiries can be directed to the corresponding author.
